# Enhancing Energy Recovery in Form of Biogas, from Vegetable and Fruit Wholesale Markets By-Products and Wastes, with Pretreatments

**DOI:** 10.3390/plants10071298

**Published:** 2021-06-26

**Authors:** Carlos Morales-Polo, María del Mar Cledera-Castro, Marta Revuelta-Aramburu, Katia Hueso-Kortekaas

**Affiliations:** 1 Institute for Research in Technology, Comillas Pontifical University, 28015 Madrid, Spain; mcledera@comillas.edu; 2 Department of Mechanical Engineering, ICAI School of Engineering, Comillas Pontifical University, 28015 Madrid, Spain; mrevuara@comillas.edu (M.R.-A.); khueso@comillas.edu (K.H.-K.); 3 Rafael Mariño Chair for New Energy Technology, Comillas Pontifical University, 28015 Madrid, Spain

**Keywords:** waste recovery, sustainable recovery, food waste, fruit and vegetable waste, biomethane, anaerobic digestion, pretreatments

## Abstract

Residues and by-products from vegetables and fruit wholesale markets are suitable for recovery in the form of energy through anaerobic digestion, allowing waste recovery and introducing them into the circular economy. This suitability is due to their composition, structural characteristics, and to the biogas generation process, which is stable and without inhibition. However, it has been observed that the proportion of methane and the level of degradation of the substrate is low. It is decided to study whether the effect of pretreatments on the substrate is beneficial. Freezing, ultrafreezing and lyophilization pretreatments are studied. A characterization of the substrates has been performed, the route of action of pretreatment determined, and the digestion process studied to calculate the generation of biogas, methane, hydrogen and the proportions among these. Also, a complete analysis of the process has been performed by processing the data with mathematical and statistical methods to obtain disintegration constants and levels of degradation. It has been observed that the three pretreatments have positive effects, when increasing the solubility of the substrate, increasing porosity, and improving the accessibility of microorganisms to the substrate. Generation of gases are greatly increased, reaching a methane enrichment of 59.751%. Freezing seems to be the best pretreatment, as it increases the biodegradation level, the speed of the process and the disintegration constant by 306%.

## 1. Introduction

The agri-food industry is, in general, one of the most exposed to the challenges and opportunities of sustainability, due to its importance, size, production and capacity [1]. One of these challenges is the generation and management of by-products and waste, inevitable and extensive on equal parts.

However, the agri-food industry is one of the major producer of wastes and by-products, both at global scale as within the primary sector. Materials from the food supply chain (FSC), whether intended or not for human consumption, can be lost, degraded, contaminated or disposed of. They are then considered food waste (FW) or food loss (FL) [2], or even Food By-Product (FBP), in case it can be reused. 

According to different estimates, around 33% of all food produced globally is lost as FW, FBP and FL [3,4]. Specifically, 90 tons of food-related waste are generated annually in the EU [5]. This means that each person generates about 76 kg of FW per year [6] at domestic scale only. But if the whole FSC is considered, this figure increases to 179 kg of FW and FL per person per year [7]. This waste generation accounts for 10% of the total food entering homes and 25% of all food processed in the FSC [8]. 

FW and FBP should not be seen as “something to be disposed of” but rather as a new useful resource, by the same industry or by others to produce other materials or by-products with higher added value, such as animal feed, fodder and compost [9]; biochemical compounds such as organic enzymes and acids [10]; new materials such as bioplastics and biopolymers [11,12,13]; energy use by producing bio-methane [14,15,16], bioethanol [17,18], biodiesel [19] or bio-hydrogen [20,21]. 

Anaerobic Digestion (AD) is a biological process in which, by the action of specific microorganisms, and under anaerobic conditions (in absence of air and oxygen), the organic matter of a substrate is transformed into biogas, a mixture, predominantly methane (CH_4_). The residue and by-products of fruit and vegetable markets are an ideal substrate due to their moisture and high organic content, as has been shown in previous research [22].

The anaerobic degradation process consists of a series of linked processes [23], each producing the substrates needed for the development of the following stage. The organic matter (OM) found in the substrate first undergoes a hydrolysis process [24]. Originally present in the form of lipids, carbohydrates and proteins, the OM is transformed in an extracellular reaction into simpler and more soluble organic compounds such as amino acids, saccharides and long-chain fatty acids (LCFA) [25]. The substrates generated in this phase then undergo acidogenesis, a stage in which volatile fatty acids (VFA) (propyonic, butyric, valeric...) and other by-products such as alcohols, NH_3_, CO_2_ and H_2_ are obtained. The next phase, acetogenesis, further digests volatile fatty acids and other by-products into acetic acid. Lastly, acetic acid is transformed by acetoclastic methanogenesis and hydrogen methanogenesis into methane [26].

**Figure 1:**
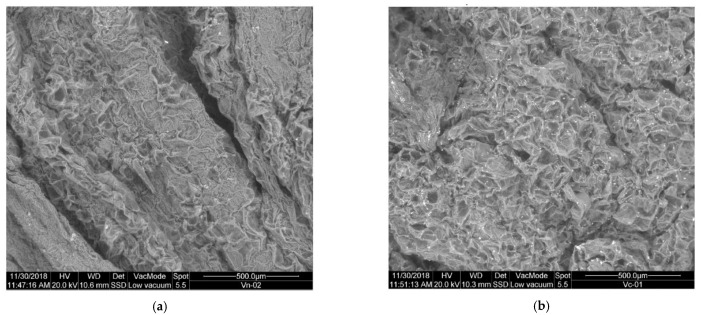
SEM micrography: (**a**) substrate V (untreated); (**b**) substrate VF (pretreated by freezing).

AD is an extremely complex process. It needs to digest all substances simultaneously so that the substrate is ready for the reactions of the next phase, and this makes the first one, hydrolysis, a limiting stage. The structure of the substrate and its accessibility to microorganisms is a key parameter for the development of hydrolysis: A dissolved, or easily soluble substrate is always more hydrolyzable than a particulate one. Particulate residues with porous structure are more easily hydrolyzable than wastes with a compact structure. For example, in terms of FW and FBP, the composition of fruit residues (whose structure is porous (see further Figures 1, 8 and 15) is dominated by simple carbohydrates (see further Tables 2–4) and problems arise with the development of hydrolysis. This effect has been shown by other authors in previous work [22] on which this study is based and demonstrate the viability of fruit and vegetable residue for anaerobic digestion, but with a pathway to improve the disintegration of particulate matter.

Substrate accessibility can be modified by physical (increased porosity and available surface area) or by chemical reactions (forming complexes with other components). This can be done through pretreatments, i.e., making pre-substrate modifications, before being introduced into the digester and digested anaerobically. The goal of pre-treatment is to adapt particulate matter, making it more accessible to microorganisms and speeding up the process of disintegration + hydrolysis, triggering subsequent digestion phases more quickly and effectively. 

Pretreatments are classified into several categories, namely, physical and mechanical; chemical; thermomechanical; and biological. The purpose of all of them is to improve the development and efficiency of disintegration + hydrolysis and increase solubilization, through different mechanisms (reduction of size and particle, increase of porosity, increase of available surface area, rupture of cell walls...) [27,28].

Physical and mechanical pretreatments seek to separate unwanted objects from OM and to reduce its size. Consequently, the surface area needed for enzymatic action as well as the contact area between substrate and microorganisms is increased [29]. When the particle size is sufficiently reduced, the performance rate of methane production can scale up to 40%. The most common methods for size reduction are grinding, crushing, pressing or screening. As an example, some researchers have achieved increases in biogas production of between 9–34% if the substrate was crushed prior to AD [29], 20% if the substrate was dilacerated [30], or 94% in both biogas and methane production if the material was previously sifted and sonicated by ultrasound [31,32]. However, an excessive reduction in particle size can result in a hydrolysis overload, and, therefore, an accumulation of VFA. This situation has been identified when the substrate is excessively crushed [33], causing a collapse in methanogenic activity. 

The main goal of this process is to hydrolyze cellulous materials, especially vegetable or lignocellulosic FBP such as fruit and vegetable wastes [27]. Experiences with the use of basic pretreatments demonstrate that the biogas generated can grow up to 170% [34], by inducing particle expansion and increasing the available surface area. Additional consequences are the dissociation of lignin and the rupture of the bonds between lignin and carbohydrates, facilitating access to them and making it more susceptible to becoming biogas [35]. However, carbohydrate intake can be overloaded, leading to inhibition of the process by acidification and accumulation of VFAs, including formation of carboxylic acids and phenolic compounds by reaction [36], hence resulting in a decrease in biogas production of up to 66% [37]. 

As practical examples in the application of mechanical pretreatments, experiences like [38] can be mentioned, in which through a screw press and screening pretreatment found an increase in biogas generation of 15%. In addition, [29] experienced an increase in methane generation of between 9% and 14% by grinding the substrate. On the other hand, through successive increases and decreases in pressure, to break cell walls, [39] experienced a 35% increase in biogas yield. Other somewhat newer pretreatments such as sonication [31] and ultrasonication [32] achieved increases in biogas generation of 94%. But not all pretreatments show positive results, for example, in the experiences of [29] pre-treating by milling the substrate, difficulties and inhibition of the process appears due to an overstimulation of hydrolysis and therefore accumulation of VFA.

The main objective of this study is, based on previous results and research on anaerobic digestion of residues from fruit and vegetable wholesale markets, determine whether pretreatments are a feasible option to improve and optimize the digestion process, the amount of biogas generated and the amount of methane it contains. All this by attacking one of the fundamental limitations detected in the process, such as the low level of degradation. A series of mechanical pretreatments, which are also easily accessible in these markets, such as conventional freezing, ultrafreezing and lyophilization, have been studied.

To achieve these objectives, an in-depth characterization of the by-products used as a substrate has been carried out, before and after being pretreated. The means of action of pretreatment has been studied and determined, and its level of effectiveness assessed. Subsequently, the generation and evolution of hydrogen has been monitored, as it is an intermediate gas that is generated in the process and then transformed into methane. It has been used as an indicator to understand whether the process (with pretreated substrate or not) has suffered inhibitions or not [22]. Also, the generation of biogas and methane has been controlled and analyzed to determine the development of the process in detail. The results obtained have been treated with statistical and mathematical analysis, which allowed the determination of the best pretreatment of the substrate to be used as a source of methane generation, and therefore contribute to meeting the objectives indicated in the European strategies [40,41,42,43].

## 2. Materials and Methods

### 2.1. Test Samples

The substrates used in this study, residues from vegetable and fruit wholesale markets (hereinafter V), are characterized by their great variability due to factors such as sales levels, consumer behaviour or seasonality. To avoid these changes and variability it has been decided to use a laboratory-prepared residue, with similar characteristics to those studied in the bibliography and to those observed by the authors. The composition is kept constant, and the influence of this residue can be optimally determined on the anaerobic process, and the effects of pretreatments on its outcome.

On the other hand, the source of microorganisms comes from the anaerobic sludge of a wastewater treatment plant equipped with an Upflow Anaerobic Sludge Blanket (UASB) reactor (hereinafter F). This source of microorganisms is one of the recommended by UNE [44] and VDI [45] standards.

### 2.2. Analytical Methods for Compositional Characterization of Substrates, Mixtures and Pretreatments

Standard procedures are used for the characterization of substrates, inoculum (sludge), and substrate + inoculum mixture introduced into the reactor, either pretreated or not. Specifically, those methods described in Standard Methods for the Examination of Water and Wastewater of the APHA (2015) [46] have been followed. All these methods and their standardized code used in substrate characterization are summarized in Table 1. The materials have been characterized in terms of:

- Physicochemical composition: Determination of the content in total solids (TS), volatile solids (VS), humidity (Hum), and Chemical Oxygen Demand (COD), also specifying total (CODt) and soluble (CODf). These two COD measures also include the solubility coefficient, which indicates the amount of COD directly accessible to microorganisms. This is a relevant indicator to analyze the results of pretreatment and whether it has enhanced the solubilization of hydrolytic enzymes.
- Organic composition: The development of a macronutritional analysis allows to determine the content of lipids, proteins and carbohydrates (LPCH), and determines the effect of the material that enters the anaerobic digestion process.
- Elemental Analysis: Determines the content in C, N, H and S, and the C/N ratio whose optimal value to ensure proper development of digestion is around 20. This ensures that there is enough carbon to digest, and that the nitrogen content is not high to cause ammonia accumulation and inhibition.
- Nitrogen content: Being nitrogen a fundamental element for digestion as nutrient and by releasing ammonia that can cause a buffer effect and reinforce the digestion process. But, at the same time, it is considered an inhibitor if high amounts of ammonia are released during digestion, causing a drastic increase in pH and the failure of the process. The analysed parameters were Total Kjeldahl Nitrogen (TKN), Organic Nitrogen (ON) and Ammoniacal Nitrogen (AN). The latter is an indicator of the formation and accumulation of ammonia. The limit for the accumulation of AN determined here is 2 mg/mL or 2 mg/g. Below this level it functions as a buffer, regulating pH; above it, it causes accumulation and subsequent inhibition.
- pH and alkalinity: pH is used as a reference of process development. The accumulation of acidic elements is shown by a decreasing pH, whereas the accumulation of ammonia is expressed with an increase in pH. Total alkalinity (TA), partial alkalinity (PA) (due to bicarbonates) and intermediate alkalinity (IA) (due to VFA) are indicators of alkalinity. A decrease in IA is indicative of excessive generation of VFA is shown with a decrease in IA and its accumulation can be verified with pH variations.

### 2.3. Pretreatment Procedure and Study of Its Influence

Given the scope of application (wholesale markets of food products) it has been decided to select easily accessible pretreatments, with machinery already present in this type of facilities. These pretreatments are conventional freezing, ultrafreezing and lyophilization.

To freeze a substrate, the sample was slowly frozen at −18 °C for 24 h. After this time, the frozen substrate (VF) is directly subjected to digestion in batch assays. For ultrafreezing, a substrate sample was introduced into liquid nitrogen for 2 min and used immediately as substrate (VU). An industrial freeze-drier is used to freeze substrates. The substrate freezes at −80 °C for 24 h and undergoes freezing vacuum, for 24 h, to extract all the contents in water. Once lyophilized the substrate (VL) is used directly.

As these are three physical pretreatments, their mechanism of action or improvement of digestion may be due to an increase in solubilization or an increase in available surface area and rupture of cell walls.

Measurement of the solubility parameter and CODf calculations have been used to analyse changes in substrate solubilization of substrates when pretreating them. If solubility is increased, the liquid fraction will contain more organic matter available to be hydrolysed and digested directly, therefore the total COD will remain constant, but the filtered COD will increase, and so will the solubility coefficient S. If there is no change in filtered COD, it means that the pretreatment has had no effect on solubility. If, on the other hand, the filtered COD is increased after being pretreated, the solubilization has improved, and therefore the disintegration + hydrolysis process will be accelerated.

SEM micrography has been used to determine whether there has been a rupture of walls, or whether the surface made accessible by pretreatment has been increased. Slow osmotic dehydration with ethanol has been chosen to prepare the samples. To do this, samples have been immersed in ethanol solutions of increasing concentration, from 30% to absolute ethanol, during 20 min each.

### 2.4. Biochemical Methane Potential Tests to Determine Anaerobic Biodegradation

The Biochemical Methane Potential (BMP) test procedure has been developed in accordance with UNE-EN ISO 11734 standard [44], which includes the measurement of biogas composition with gas chromatography. Manometric methods are used to calculate the amount of gas generated. The procedure described in VDI-4630 [45] has been followed to transform the pressure measurements into generated gas flow.

The test conditions are equivalent to full-scale level. The digestion takes place at mesophilic temperature (37 ± 1 °C), with a 1:3 *m*/*v* ratio between between residue (substrate) and UASB sludge of sewage treatment substrate (inoculum). Hence, 100 g of fruit and vegetable residue have been digested for every 300 mL of UASB sludge.

The quantity of gas generated and its composition in methane and hydrogen are measured by gas chromatography on a daily basis, as described in the Standard Methods for the Examination of Water and Wastewater of the APHA (2015) [46]. From this, the curves of biodegradability of the substrate are obtained, as well as the generation of methane and hydrogen and their content in biogas.

The process and the variables that affect it can be inferred from the joint analysis of biodegradability curves, methane and hydrogen generation, as well as the characterization of the substrate before and after the BMP test. 

In this case, hydrogen is especially relevant, as it is an intermediate gas that starts to be generated in the early phases of the process, and gradually disappears as it is transformed into methane. The development of hydrolysis directly depends on the rate of generation of hydrogen, whereas the ability of the process to complete subsequent phases depends in turn of the transformation of hydrogen into methane.

### 2.5. Statistical Analysis of Results

The tests are repeated in 21-test runs, resulting in a significant number of curves. An ANOVA statistical analysis with a 95% confidence level accompanied by their respective DMS and Tukey contrasts, is performed to establish whether all curves can be considered equal, or if the dispersion between them is large enough to indicate no relationship.

### 2.6. Mathematical Determinations and Adjustments

In addition, the results of the curves obtained have been treated mathematically. Considering methane generation curves as a first-order kinetics, according to the Veeken and Hamlers [25] process description, the disintegration constant (k_dis_) can be obtained and is directly related to the ability to be treated by digestion anaerobia and the speed of the process. Similarly, the maximum amount of methane expected for the substrate can be obtained by adjusting by least-squares the methane generation curves with the reduction experienced in the COD, according to the [47] procedure. In addition, by reducing the COD content, as set out in [47], the percentage of substrate that has been biodegraded can be determined, which is directly related to the scope of the process.

## 3. Results and Discussion

The objective of this study is to determine the influence of certain pretreatments on the anaerobic digestion process of substrate V. Therefore, all results are compared with previous experiences of anaerobic digestion of residue V, already published in [22], by the obtained numerical data. However, the results obtained in that previous investigation [22] are briefly presented in the following section.

### 3.1. Short Review of Previous Anaerobic Digestion Results of Substrate V

As for its composition, residue V is presented as a substance mostly made up of carbohydrates, with a high solubility (24.09%) and with resistance to pH changes due to its alkalinity (TA 5.830 mg CaCO_3_/g) (see further Tables 5, 9 and 13). The high carbohydrate content is positive as it produces a stable digestion, however, a large majority of them are simple carbohydrates like lignocellulose, which can compromise anaerobic development due to their low biodegradability. 

During the study of the process, it is determined that no inhibition takes place and that digestion is stable (as could already be inferred by carbohydrate content and alkalinity), although it is incomplete as a high methane content is not reached and the biodegradation level of the substrate is low (only 16.045% of the degradable material of residue V is degraded). Precisely because of this low degradation it is an interesting residue to be pretreated and thus improve its level of degradation.

The anaerobic digestion process occurs in two stages. First, one in which the already solubilized organic matter is digested, and the second, in which the rest of the organic matter that is encapsulated begins to be digested. Therefore, it is an interesting residue to be treated by physical pretreatments like those studied. One of the main lines of action of these pretreatments is the improvement of the solubilization of organic components by breaking cell walls of particulate and encapsulated matter.

Specifically, 100 g of digested V substrate produce 913.282 NmL of biogas, of which 289.333 NmL correspond to methane. The substrate disintegration constant is 0.200 days^−1^, degrading only the 16.045% of the substrate (Table 2, Table 3 and Table 4).

### 3.2. Results for Freezing Pretreatment (VF)

#### 3.2.1. Course of Action of Pretreatment

##### Changes in Substrate Characterization When Freezing

Once the residue V has been frozen, it is characterized to determine whether there have been changes in its composition that may favour any variation in degradation. Table 5 shows these results. In addition, the results of the characterization of the inner mixture of the reactor are also shown before being digested and after the completion of the BMP tests that allow to determine the development of the anaerobic process according to the evolution of the parameters.

As seen when comparing the characterization results of substrate V (untreated) and VF substrate, there are no apparent compositional differences, except in terms of solubility and alkalinity. Solubility is increased by +32.918%, so there is more OM directly available for microorganisms and a greater conversion and generation of methane is expected, with an improved biogas enrichment. It can therefore be assumed that one of the means of action of the freezing pretreatment on residue V is the improvement in solubility.

In terms of alkalinity, when pretreating the substrate, it is reduced by −53.516% of both IA and TA. That is, the substrate becomes more vulnerable to changes in pH and therefore to the release of VFAs that occurs when the substrate V (carbohydrates rich) is degraded. 

As for the results of the characterization before and after BMP assays, it is observed that the process has developed correctly by reducing TS and vs. and a large amount of COD, especially the soluble one. The released AN reaches values below the accumulation limit (2 g/L), so it is not in sufficient concentration to cause inhibition and it will act as a buffer in the face of possible acidification. In terms of alkalinity, this is reduced in any case, with a reduction of −64.139% in IA, indicating that a VFAs release has occurred. However, pH values indicate that it has not been high enough to cause acidification and consequent inhibition.

##### Changes in the Outer Structure of the Substrate When Freezing

Figure 1 shows the images obtained in SEM micrography for the substrate before freezing V (a) and once frozen VF (b).

As noted, there is a big difference between the external structure of the untreated V substrate, and the VF substrate once frozen. A clear rupture of the outer membranes is observed with a noticeable increase in porosity, so that there is a greater surface area available for the action of hydrolytic enzymes, and therefore COD becomes more accessible. It is therefore assumed that another of the routes of action of freezing pretreatment on substrate V is the rupture of outer membranes, increasing the porosity and surface available for the access of microorganisms.

#### 3.2.2. Biogas Production

**Figure 2:**
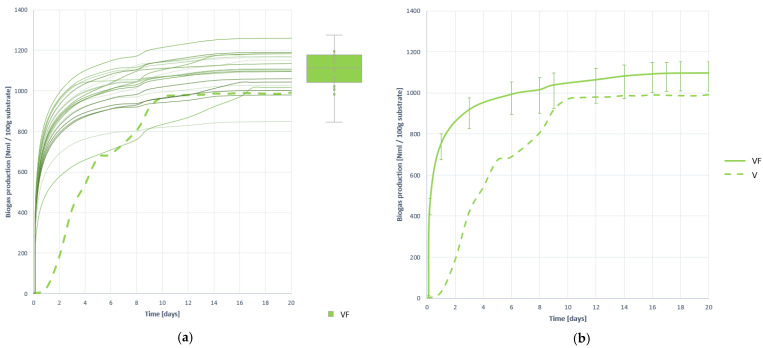
Biogas production curves obtained when digesting substrate VF: (**a**) all curves; (**b**) mean curve.

Figure 2 shows all the biogas generation curves obtained when treating VF substrate, and the average generation curve, both compared with the average or mean generation curve of the untreated V residue. Table 6 presents the numerical results along with the most relevant descriptive statistics for its comparison with other cases.

As observed, biogas generation is much faster with the VF substrate than with substrate V, so the effect of pretreatment has been very positive in terms of process speed. However, overstimulation of the process can cause excessive release of VFAs that cause acidification, although as studied in the characterization of the mixture in digesters, this is not likely. In quantitative terms, the effect of pretreatment has been very positive, increasing by 20.20%, reaching an average generation value of 1097.469 (±6.094%) NmL. Likewise, in terms of stability there is an improvement in the coefficient of variation (CV) between the gas generation curves is lower.

#### 3.2.3. Methane Production

**Figure 3:**
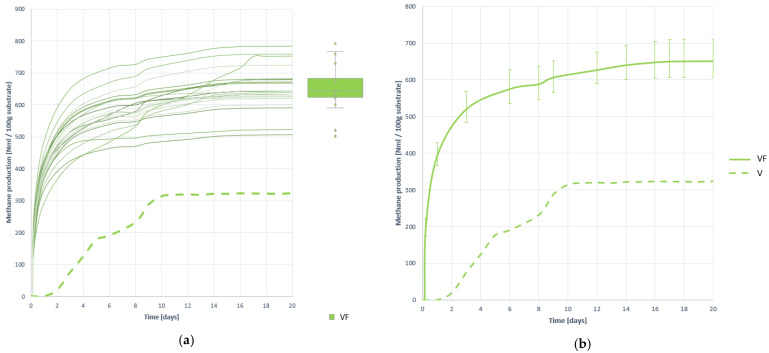
Methane production curves obtained when digesting substrate VF: (**a**) all curves; (**b**) mean curve.

In terms of methane production, the effects of pretreatment are even more effective. As seen in Figure 3 not only a huge increase in the speed of the process is achieved, but also a great growth in the total amount of methane generated.

The total average amount of methane generated with 100 g of VF substrate is 651.319 (±7.790%) NmL, i.e., 125.370% higher than methane generated by untreated residue V. In addition, it is not only gained in terms of generation, but also in process stability, the variability and dispersion between curves being much lower with the VF substrate than with the substrate V (without pretreating), as extracted through a visual analysis of the curves and analytically with the VF. It is therefore assumed that the effect of pretreatment has been very positive in terms of the acceleration and harnessing of methanogenesis, and its stability.

#### 3.2.4. Methane Proportion in the Generated Biogas

**Figure 4:**
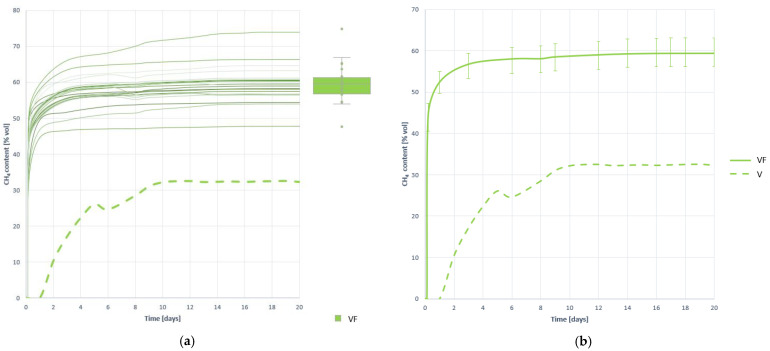
Methane proportion curves contained in the generated biogas obtained when digesting substrate VF: (**a**) all curves; (**b**) mean curve.

When analyzing the proportion of methane in biogas, i.e., methane enrichment, it is concluded again that the effect of pretreatment is very positive. As observed in Figure 4 methane is detected in biogas practically from the beginning of digestion, which is indicative of a good speed of hydrolysis and a better accessibility of the substrate to microorganisms. As from Table 7 is extracted, the average methane ratio in biogas is 59.438 (±5.838%) %CH_4_, an increase of 85.743% over the proportion of average methane recorded in substrate V without pretreatment.

The value, very close to 60%, indicates that the process has been developed in a deep and stable way. In fact, the improvement in pretreatment is not only associated with an increase in the percentage of methane, but also with the stability of the generation, which was greatly increased with respect to that recorded in the untreated V residue tests (comparing the coefficient of variation).

The shape of the curves, reaching stabilization very soon, indicates that the process has developed very quickly. To confirm that the process has quickly and fully developed, and that there is no inhibition derived from the increase in speed and overstimulation of hydrolysis, the generation and evolution of H_2_ created is studied.

#### 3.2.5. Hydrogen Production

**Figure 5:**
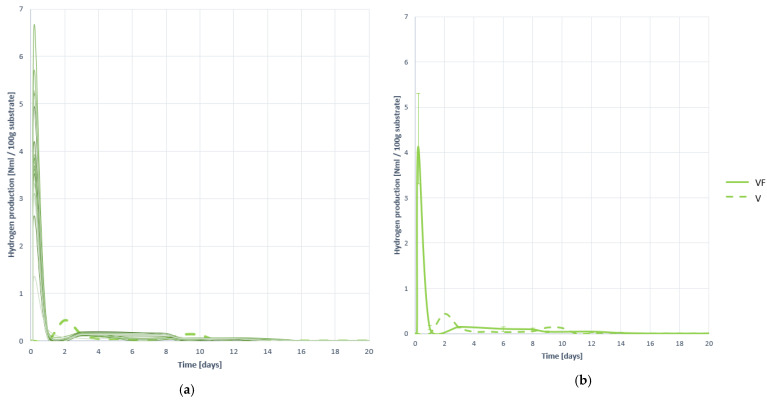
Hydrogen production curves obtained when digesting substrate VF: (**a**) all curves; (**b**) mean curve.

The H_2_ evolution curves represented in Figure 5 show that H_2_ is generated at high speed, peaked much higher than detected with the untreated V substrate, and before day 1. The H_2_ begins to disappear at a very high speed, without remaining. It is therefore understood that the high speed of the process does not cause any inhibition by excessive release and accumulation of VFAs, but that the process develops quickly and correctly.

It is observed that the phenomenon of digestion in two phases that occurs with the substrate V without pretreating disappears completely, as a single hydrogen peak appears. It is therefore assumed that pretreatment has been beneficial and has facilitated solubility, by not having to digest in two stages.

#### 3.2.6. Hydrogen Proportion in the Generated Biogas

**Figure 6:**
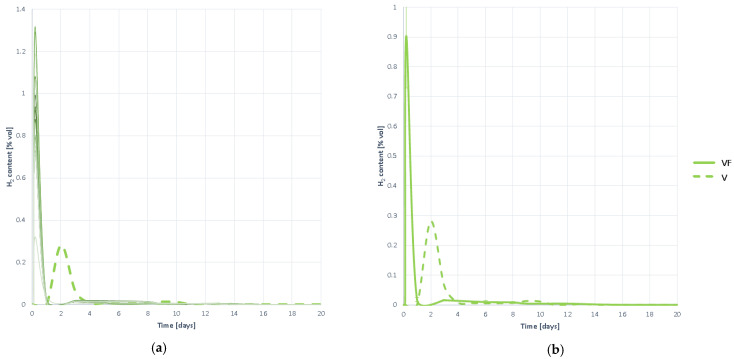
Hydrogen proportion curves contained in the generated biogas obtained when digesting substrate VF: (**a**) all curves; (**b**) mean curve.

The same conclusions are drawn from the hydrogen ratio curves in biogas, represented in Figure 6, as those developed after the analysis of hydrogen production. 

A large amount of hydrogen begins to be detected in a very early time, and in large quantities. This indicates, not only that the process speed is high, but that hydrolysis is deep, with a large amount of H_2_ being detected. The rate of elimination of H_2_ is also rapid, and hydrogen does not remain, so there is no inhibition and hydrogenotropic methanogenesis develops smoothly.

#### 3.2.7. Evaluation of the Evolution of the Digestion Process

**Figure 7:**
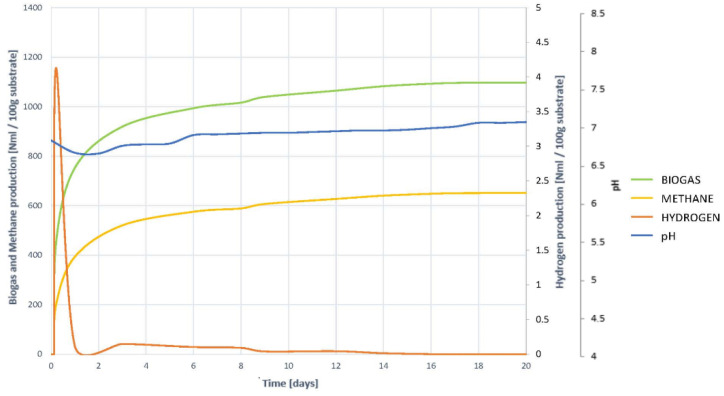
Evolution of the digestion process of substrate VF. Comparison of the generation of biogas, methane and hydrogen together with the evolution of pH.

To understand the development of the AD process, and to check the hypotheses of the non-existence of inhibition, it is necessary to study the joint evolution of the biogas, methane and hydrogen generation curves, with the change in pH, shown in Figure 7.

It is observed that the generation of both methane and biogas, on the one hand, and hydrogen, on the other, begins at high speed from the first moment. Hydrogen generation and elimination coincides with the steepest slope phase in biogas and methane generation curves. It is therefore understood that hydrogenotropic methanogenesis occurs during the first few days. Once H_2_ has completely disappeared, although a very small amount remains, the generation of both gas and methane are slowed down, without fully stopping. This indicates that other gases continue to be generated, and methanogenesis does not halt, so it occurs acetoclastly, and therefore it is understood that there is no inhibition by accumulation of VFAs. In such a case, an increase in gas generation would be seen, but methane levels would remain constant.

The evolution of pH corroborates all the hypotheses inferred. A slight acidification occurs during the first days, the result of the natural generation of VFAs, and coinciding with the presence of H_2_. Once all H_2_ has been transformed into CH_4_, light acidification is progressively recovered, so there is no accumulation of VFAs, despite overstimulation of hydrolysis.

#### 3.2.8. Mathematical Analysis and Adjustment of the Digestion Process

The results of the mathematical analysis and the adjustment of the digestion process are shown in Table 8. The generation, both the maximum obtained and the theoretical expected, are much higher when digesting the VF substrate than substrate V. Both generations increase by 118.318% and 101.646% respectively. This indicates that the process has taken place in greater depth, and that methanogenesis has developed with a higher level, motivated by a better accessibility to the substrate, and by an improvement in solubility. The disintegration constant is greatly increased by 306.500%, which clearly indicates that hydrolysis is accelerated and stimulated by improvements introduced with pretreatment. Improvements in methane levels and process stability are due to an increase in the level of degradation of the substrate, which degrades by an additional 34.403% when pretreating by freezing substrate V.

It is therefore concluded that the freezing pretreatment of substrate V is beneficial by increasing the solubility of the substrate and causing a rupture of external membranes that greatly increase the surface area available for the accessibility of microorganisms. This facilitates, accelerates and stimulates hydrolysis, generating much more methane and in greater proportion, through a more stable process, than in the case of not pretreating the substrate.

### 3.3. Results for Ultrafreezing Pretreatment (VU)

#### 3.3.1. Course of Action of Pretreatment

##### Changes in Substrate Characterization when Ultrafreezing

Once the substrate V has been subjected to ultrafreezing, it is characterized to determine if there are changes in the composition that may affect the anaerobic degradation of the pretreated substrate VU. These results are shown in Table 9 which collects the results of the characterization of the Vu substrate compared to those obtained for the substrate V without pretreatment. Characterization data from the reactor’s inner mixture before and after the end of BMP tests are also provided to determine possible changes in anaerobic degradation.

Comparing the characterizations of the untreated V substrate and the VU (pretreated) no compositional differences are seen, except for changes in alkalinity. By ultrafreezing the substrate V, alkalinity, both IA and TA are reduced by −31.046%, thus making the substrate more sensitive to sudden variations of pH caused by acid accumulation. It should then be noted that there is no excessive release of VFAs during the early stages of the process that may lead to acidification with the consequent inhibition of the process.

In this case, there are no changes in the solubility of the substrate when pretreated. This is understandable as, being rapid freezing, an escape from COD cannot occur, becoming soluble. ultrafreezing pretreatment cannot then be considered to cause changes in substrate solubility. As for the development of the process, it is observed that it occurs normally. A reduction of TS and vs. occurs, with a significant elimination of COD, especially the soluble part. There is also a slight release of AN, falling below the inhibition limit, so it is expected that, in case of acidification, it will act as a buffer. In terms of alkalinity, especially IA is reduced, indicating that there has been a significant release of VFAs. However, it does not appear to have been important enough to generate inhibition, as the value of the start and end pH remains virtually constant, being higher than the end of the process.

##### Changes in the Outer Structure of the Substrate When Ultrafreezing

**Figure 8:**
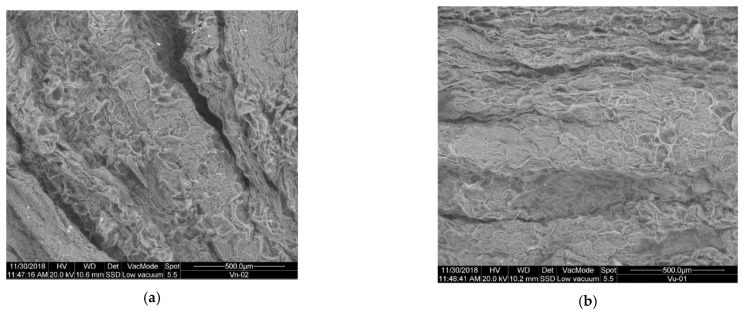
SEM micrography: (**a**) substrate V (untreated); (**b**) substrate VU (pretreated by ultrafreezing).

Figure 8 shows the images obtained in SEM micrography for the substrate before ultra-freezing V (a) and once ultra-frozen VU (b).

Comparing the structure of the V substrate without pretreatment and subjected to ultrafreezing (VU), it shows changes in the structure, but not as pronounced as in the case of slow freezing. It is observed that the outer walls look stiffer or crystallized, with a more fragile and thin appearance. In addition, there is a slight increase in pores and their size, without excessive change. It can then be assumed that the ultrafreezing pretreatment of substrate V causes a weakening of the outer membranes, and a minimal increase in porosity, generating a greater surface area available for the action of microorganisms. In any case it is not comparable to the effect of slow freezing.

#### 3.3.2. Biogas Production

**Figure 9:**
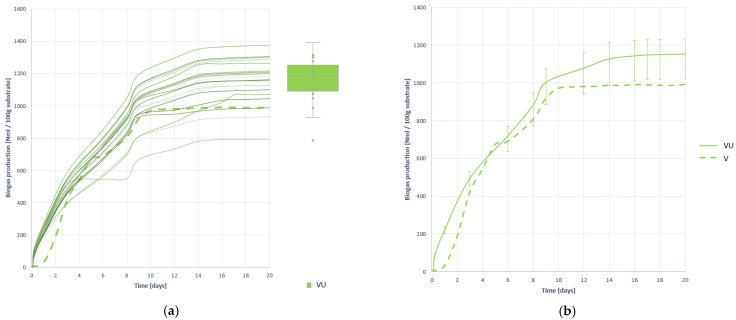
Biogas production curves obtained when digesting substrate VU: (**a**) all curves; (**b**) mean curve.

Figure 9 represents all biogas production curves obtained with BMP assays, and in Table 10 the quantitative values of production and descriptive statistics most relevant to their analysis.

By comparing the biogas generation of the VU substrate with that of the untreated substrate V, it can be determined that the effect of pretreatment has been positive in terms of gas generation, by increasing the gas generation by 26.341% compared to that obtained per untreated V. Specifically, 1153.490 (±8.667) NmL is obtained per 100 g of V residue treated with ultrafreezing.

In terms of gas generation speed, a slight increase is observed, but it is not comparable to the speed gain achieved with VF. The improvement in speed will then be determined by the calculation of the disintegration constant.

Pretreatment has also been beneficial for the stability of generation, as can be seen in the generation curves and statistically shown with the ANOVA study and the coefficient of variation (CV).

#### 3.3.3. Methane Production

**Figure 10:**
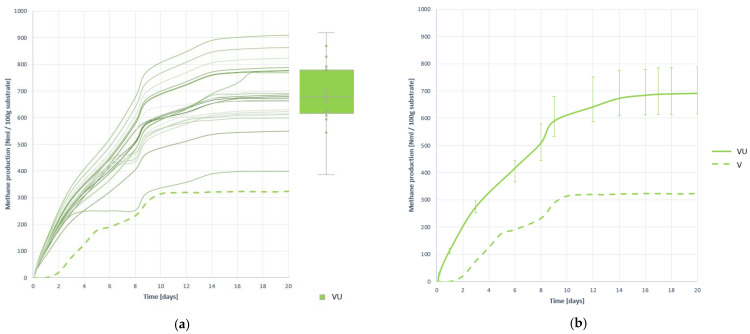
Methane production curves obtained when digesting substrate VU: (**a**) all curves; (**b**) mean curve.

As seen in Figure 10, which shows the methane generation curves with the substrate VU and compared to the generation of the substrate V without pretreatment, the effect of pretreatment has been positive, in terms of generation level and process speed.

Pretreatment of substrate V by ultrafreezing achieves an average CH_4_ generation of 690.123 (±12.220) NmL, an increase of 138.797% over V methane generation. It is also observed that the curves grow faster. It can even be assumed that methanogenesis develops deeper as generation continues to grow until day 16, while untreated V digestion stabilized on day 10. In terms of stability, ultra-freezing pretreatment is also effective, as they are the most convergent curves, as demonstrated by the lower CV value.

#### 3.3.4. Methane Proportion in the Generated Biogas

**Figure 11:**
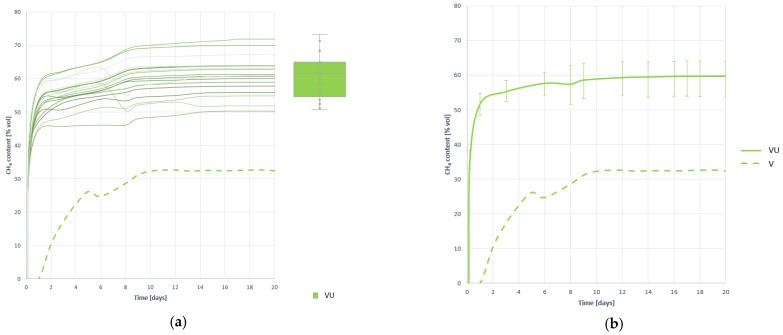
Methane proportion curves contained in the generated biogas obtained when digesting substrate VU: (**a**) all curves; (**b**) mean curve.

Figure 11 shows the methane ratio curves in the biogas generated when digesting the ultrafreezing V substrate (VU). 

As observed, methane is detected in large amounts from the beginning, indicating that the level of methanogenesis has been improved, mainly motivated by the improved access of microorganisms to the substrate. Regarding the methane enrichment of the biogas produced by V, the effect of pretreatment has been very positive. The proportion of methane in biogas is increased by 86.612% compared to untreated V digestion, reaching an average ratio of 59.715 (±8.369) %CH_4_ (Table 11).

The shape of the curves, in which stability is quickly achieved, indicates that the process has developed rapidly without inhibition. This, coupled with the value close to 60% (indicative of stability) and that acidification by accumulation of VFAs according to the initial and final pH values is not likely, rule out the possibility of inhibition. However, the evolution of H_2_ during the process is being studied to rule it out with complete reliability.

#### 3.3.5. Hydrogen Production

**Figure 12:**
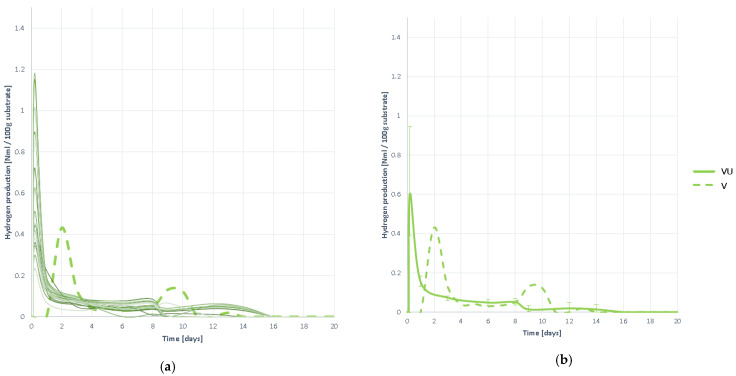
Hydrogen production curves obtained when digesting substrate VU: (**a**) all curves; (**b**) mean curve.

In Figure 12 the H_2_ evolution curves are presented during the degradation process of the substrate VU. It is observed that hydrogen generation begins rapidly, peaking at the first moments of digestion, and beginning to disappear at a high rate thereafter.

Hydrogen stays in the reactor for a long period, but in very small amounts, so there is a slight but practically negligible slowdown in hydrogenotrophic methanogenesis. Acidification inhibition can then be considered ruled out, although there is minimal slowdown. However, the evolution of H_2_ proportion in biogas should be studied and a joint analysis of all curves should be performed.

#### 3.3.6. Hydrogen Proportion in the Generated Biogas

**Figure 13:**
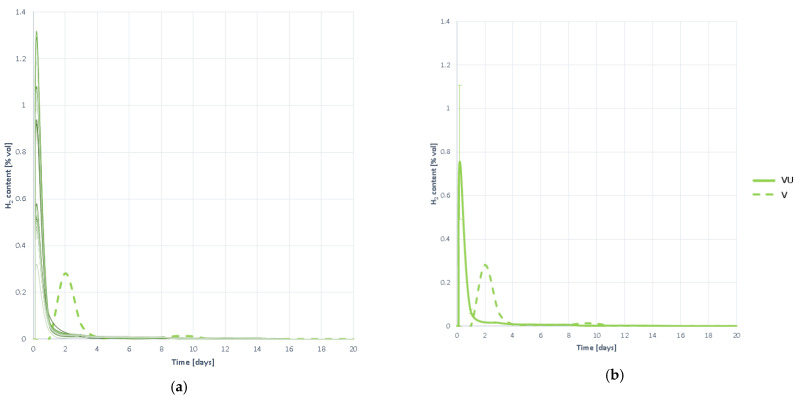
Hydrogen proportion curves contained in the generated biogas obtained when digesting substrate VU: (**a**) all curves; (**b**) mean curve.

The hydrogen content curves of the generated biogas (Figure 13) yield the same conclusions as hydrogen generation curves. This is seen in the early moments of digestion and disappears at a very high rate so that it is virtually undetected in the following days. It can therefore be concluded that there is no inhibition by acidification, in any case a slight slowdown but negligible.

#### 3.3.7. Evaluation of the Evolution of the Digestion Process

**Figure 14:**
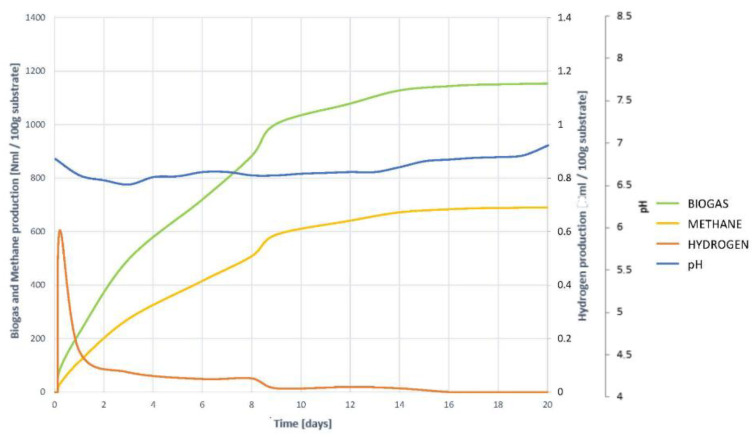
Evolution of the digestion process of substrate VU. Comparison of the generation of biogas, methane and hydrogen together with the evolution of pH.

By analysing together, the methane, biogas and hydrogen generation curves, with the evolution of pH, a clear understanding of the development of the process con be achieved (Figure 14).

It is clearly observed how H_2_ appears in the early moments of digestion and this coincides with the area of greatest slope in the generation of biogas and methane. H_2_ begins to disappear and maintains the high rate of biogas and methane generation. The elimination of H_2_ slows down, and so does the production of biogas and methane. It is then understood that at this time there is a slowdown in hydrogenotrophic methanogenesis, and as methane production does not cease, it continues to occur via the acetoclastic pathway. Once all hydrogen disappears, a spike occurs in the generation of methane and gas, to end up stabilizing.

The evolution of pH corroborates what is inferred, as while H_2_ remains, lower pH levels remain too. Once the H_2_ disappears, the pH increases. It is then understood that a slight accumulation of VFAs occurs, which slows down hydrogenous methanogenesis, but does not accumulate as acetoclastic methanogenesis occurs normally. Once the methanogenesis is complete, and therefore the VFAs disappear, the pH is recovered, and the process concludes normally. This is one of the causes, in addition to the lower accessibility and solubility, of a slower process than in the case of degradation of the pre-treated residue by slow freezing.

#### 3.3.8. Mathematical Analysis and Adjustment of the Digestion Process

Table 12 summarizes this information. The generation of methane, both the maximum obtained and the theoretical expected, are higher in the case of pretreated substrate V by ultrafreezing (VU) than when it is digested in its natural state. An increase of respectively 130.260% and 101.646% is seen. This indicates that the process has developed with greater depth, mainly motivated by the small best in accessibility to the substrate. The disintegration constant is increased by 42.500%, indicating that hydrolysis is accelerated and stimulated by improvements introduced with pretreatment. In any case the improvement is less than that obtained with VC. The level of degradation of the substrate is also increased, which corroborates the high levels of methane obtained and the enrichment of biogas.

It is therefore concluded that ultrafreezing pretreatment of VU is beneficial by causing an increase in biogas generation and methane enrichment, based on an acceleration of hydrolysis and the disintegration process. This is motivated by an improvement in accessibility to the substrate, as the outer membranes are weakened.

### 3.4. Results for Lyophilization Pretreatment (VL)

#### 3.4.1. Course of Action of Pretreatment

##### Changes in Substrate Characterization when Lyophilizating

Table 13 presents the characterization results of the untreated substrate V and VL pretreated by freeze-drying. Also shown are the results of characterization of the inner contents of the digester before and after the end of the BMP test, to determine whether, from the point of view of composition, there has been a change that affects the process.

Comparing the characterizations of V and VL, it is appreciated that with the lyophilization pretreatment there have been significant changes in the moisture content, alkalinity, and solubility of the substrate. The most noticeable change that occurs in the drastic reduction of humidity, which has been reduced by −98.805% in VL compared to V. Solubility, on the other hand, is increased by 96.762%. It is therefore to be expected that the process will develop at a slow rate due to the low moisture content, but once the substrate is well mixed with UASB sludge, there will be a large increase in methane generation due to the increase in solubility. It can then be assumed that one of the pathways of action for pretreatment is the improvement of solubility, although a high speed of hydrolysis by reducing moisture is not to be expected. For its part, the alkalinity of both IA and TA is reduced by −28.816%, making the pretreated substrate less resistant to pH changes by accumulation of VFAs, so it will be a determining factor in the development of the process.

##### Changes in the Outer Structure of the Substrate when Lyophilizating

**Figure 15:**
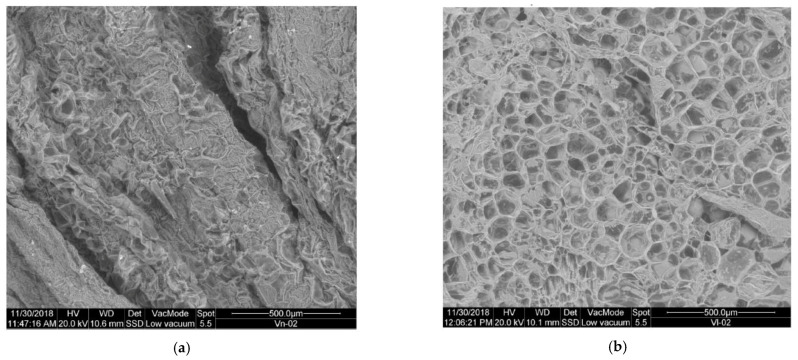
SEM micrography: (**a**) substrate V (untreated); (**b**) substrate VL (pretreated by lyophilization).

Figure 15 shows the images obtained in SEM micrography for the substrate before lyophilization V (a) and once lyophilized VL (b). Lyophilization, also known as freeze-drying pretreatment, causes major changes in the outer structure of the substrate, which becomes very porous, and the walls become thin. It can therefore be assumed that another of the pretreated means of action is increased porosity, reduced wall thickness and increased surface area available for microorganisms.

#### 3.4.2. Biogas Production

**Figure 16:**
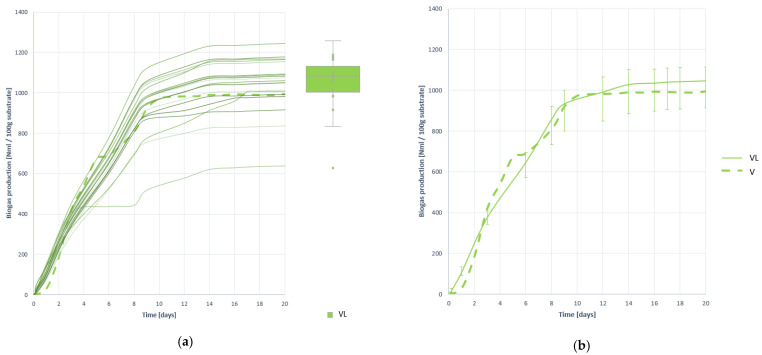
Biogas production curves obtained when digesting substrate VL: (**a**) all curves; (**b**) mean curve.

Once the substrate VL has been anaerobically digested, the biogas generation curves shown in Figure 16 are obtained. Pretreatment is shown to produce a small increase in biogas generation of 14.621%, reaching an average generation of 1046.492 (±8.564) NmL per 100 g of V residue subjected to lyophilization.

In terms of process rate, there is no improvement, as expected given the low humidity remaining after pretreatment. However, if there is an increase in the stability of the generation, being the most convergent curves as demonstrated by the graphs and descriptive statistics of Table 14.

#### 3.4.3. Methane Production

**Figure 17:**
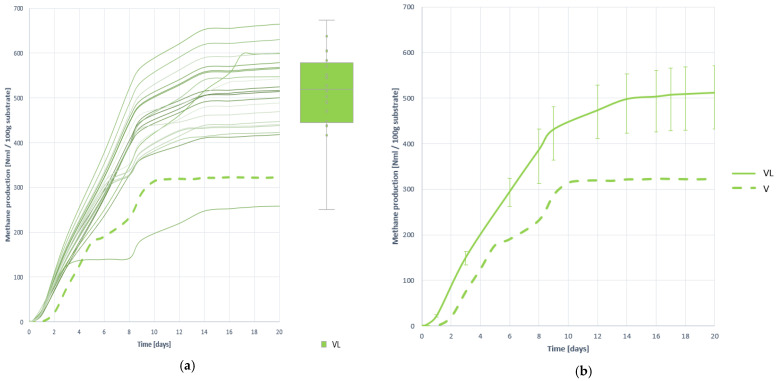
Methane production curves obtained when digesting substrate VL: (**a**) all curves; (**b**) mean curve.

Figure 17 shows all methane generation curves obtained in BMP assays, and Table 14 the most relevant descriptive statistics for their study.

It is appreciated that the effect of pretreatment is very positive in terms of methane generation, increasing by 77.044% and achieving an average generation of 511.657 (±13.333) NmL with 100 g of substrate VL. There is also a noticeable increase in the rate of methane generation, despite not being seen in biogas production, and process stability. Therefore, a rapid growth in the proportion of methane in biogas will be expected.

#### 3.4.4. Methane Proportion in the Generated Biogas

**Figure 18:**
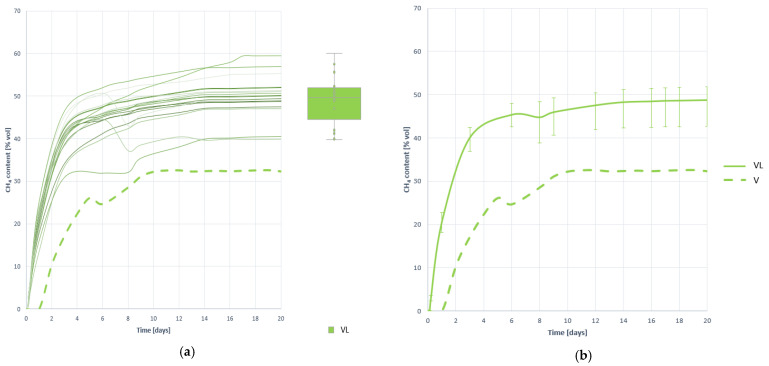
Methane proportion curves contained in the generated biogas obtained when digesting substrate VL: (**a**) all curves; (**b**) mean curve.

Figure 18 shows the evolution curves of the methane ratio in biogas by digesting 100 g of VL substrate. As noted, methane is beginning to be detected in large numbers from the outing, indicating that the level, stability and development of methanogenesis has been improved, mainly motivated by improved solubility and better access of microorganisms to the substrate. The effect of pretreatment has been very positive, increasing by 52.378% the amount of methane present in biogas, reaching an average value of 48.761 (±8.359) %CH_4_ (Table 15).

The shape of the curves indicates that the process has developed quickly, although not at the same speed as in the other two pretreatments (VF and VU). It can be assumed that the process develops without inhibition, as the methane content is rapidly increased without excessive pH changes between the initial and final values. However, the study of the development of H_2_ is necessary to verify this.

#### 3.4.5. Hydrogen Production

**Figure 19:**
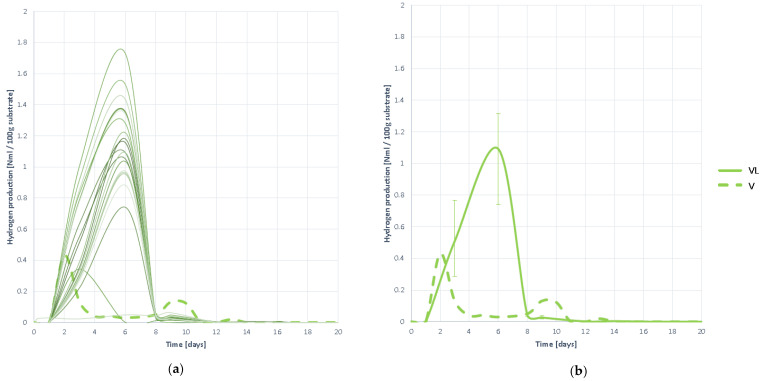
Hydrogen production curves obtained when digesting substrate VL: (**a**) all curves; (**b**) mean curve.

The production curves of H_2_ are shown in Figure 19. It is observed that hydrogen begins to grow slowly until it peaks on day 6 and begins to disappear at a high rate thereafter. It is appreciated that the rate of hydrogen generation is much slower than that of elimination. This confirms that hydrolysis occurs at a very slow rate, as expected from the low moisture content. Despite being slow, hydrolysis is deep, motivated by the action of pretreatment in accessibility and solubility, and therefore high H_2_ generation values are obtained.

Once the maximum production of H_2_ is reached, it is eliminated at an adequate speed, without further H_2_ being detected, so it is understood that, by not remaining in the reactor, there is neither inhibition nor slowing of methanogenesis of any kind.

#### 3.4.6. Hydrogen Proportion in the Generated Biogas

**Figure 20:**
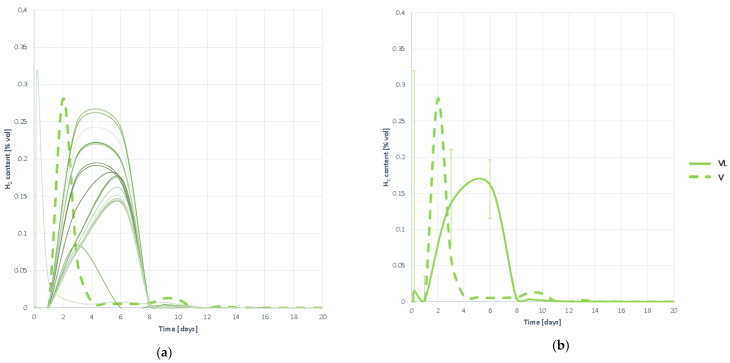
Hydrogen proportion curves contained in the generated biogas obtained when digesting substrate VL: (**a**) all curves; (**b**) mean curve.

Hydrogen ratio curves in biogas yield the same conclusions as generation curves. As shown in Figure 20 hydrogen begins to be detected on day 1 and grows slowly until it reaches a maximum several days later. 

It is then removed at a fast speed so that H_2_ is not detected again. It is therefore assumed that hydrolysis occurs at low speed, as the evolution of H_2_ is slow, but once the maximum is reached, the methanogenesis occurs at high speed, and without inhibition as not H_2_ remains over time.

#### 3.4.7. Evaluation of the Evolution of the Digestion Process

**Figure 21:**
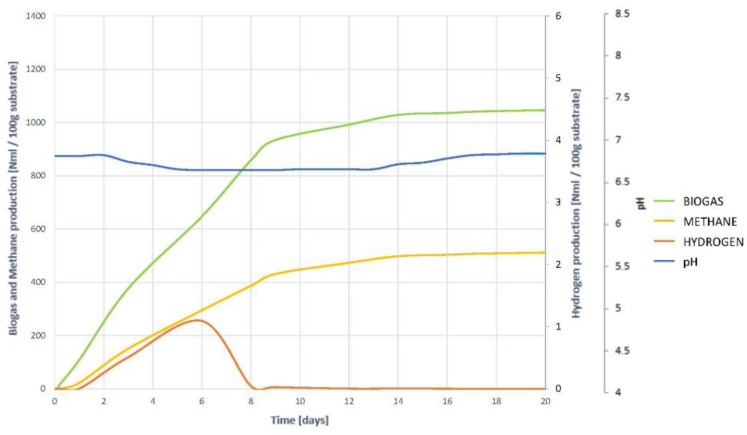
Evolution of the digestion process of substrate VL. Comparison of the generation of biogas, methane and hydrogen together with the evolution of pH.

The joint analysis of the hydrogen, methane, biogas and pH evolution curves (Figure 21) allows to determine the depth of the AD development of the substrate VL.

Hydrogen is observed to start forming very slowly, as do biogas and methane. The rate of generation of biogas and methane remains constant while hydrogen is generated and reduced, so, at first, intense acetoclastic methanogenesis occurs, which, even if H_2_ is generated, they complement each other. Precisely because of this intense acetoclastic methanogenesis there is no accumulation of VFAs in the early stages of the process.

Once the H_2_ completely disappears, the methane generation slows down, but without stopping, so methanogenesis and the acetoclastic part of it continues. As soon as the generation of biogas and methane stops, the pH begins to increase, thereby any trace of acidification disappears.

It is therefore concluded that the effect of freeze-dried pretreatment slows hydrolysis. Due to this slowness, the generation of VFAs is perfectly compensated with its elimination, not causing acidification. Once supplemented with hydrogenotropic methanogenesis, any possibility of acidification disappears and maintains a high content and generation of methane, making the process stable and durable.

#### 3.4.8. Mathematical Analysis and Adjustment of the Digestion Process

The expected and maximum expected theoretical methane generation are, in any case, higher than those determined for substrate V without pretreatment. There is an increase of 78.713% and 78.551%. This indicates that, despite the slowness, the process has developed in depth, with higher levels of degradation, motivated by improvements introduced by freeze-dried (improvement in solubility, increased surface area available for microorganisms and their accessibility). The disintegration constant is 35.500% lower, which again shows that freeze-dried substrate V slows hydrolysis. However, as solubility is significantly increased, the stability and development of the process are assured and occur normally. The level of degradation of the substrate is increased by 30.302%, which corroborates the high levels of methane obtained, despite being the slowest hydrolysis. Table 16 summarizes this information.

It is therefore concluded that the lyophilization pretreatment of substrate V is beneficial by increasing the solubility of the substrate and causing a huge increase in porosity. However, moisture is drastically reduced, and this leads to an important slowdown in hydrolysis. The increase in the solubility and accessibility of the substrate, coupled with the slow hydrolysis generates a synergy, compensated for the release of VFAs, eliminating it at a correct rate that ensures that they do not accumulate. This results in higher methane content and biogas being more enriched in methane than in the case of not pretreating it.

Table 17 shows the effect of pretreatments on substrate V, in terms of increased generation of biogas, methane, hydrogen and their contents.

## 4. Conclusions

With the study carried out, it has been shown that a highly complex procedure that improves biogas production is not necessary. Thus methane production can be implemented, with affordable processes, in any facility with WWTPs. The by-products and residues of fruit and vegetables generated in large wholesale markets, are suitable for anaerobic treatment, generating biogas with it. It is a porous residue, enriched in carbohydrates, and the digestion process is stable without inhibitors. Its biogas generation potential is 91.282 (±14.436%) NmL per 100 g of digested substrate, with a methane content of 32.252 (±12.051%) %CH_4_. It is worth mentioning that the process of digestion occurs in two phases, first by digesting the already solubilized organic matter, and secondly the particulate or encapsulated organic matter. The level of degradation of the substrate is very low, only 16.045%. There is then an open path to improve the digestion process, further degrading the substrate and obtaining a higher methane content, through pretreatment.

A mechanical pretreatment of the substrate can result in a higher than 50% increase in methane production, and thus enhance the added value of the process. The influence of different mechanical pretreatments, which modify the structure of the substrate, and whose technologies are easily accessible in the study facilities, have been studied: Freezing, ultrafreezing and lyophilization. The studied pretreatments (freezing, ultrafreezing and lyophilization) were those that do not offer much technical difficulty and are easily accessible in these agroindustrial facilities.

Freezing pretreatment increases the solubility of the substrate (+32.918%) and causes a rupture of external membranes that greatly increase the surface area available for the access of microorganisms. This facilitates, accelerates and stimulates hydrolysis, generating much more methane and in greater proportion, through a more stable process, than in the case of no pretreatment of the substrate. Some 20.200% more of biogas is generated with a much higher methane content of 59.438%.

Ultrafreezing pretreatment, although it does not produce significant changes in substrate solubility, slightly weakens the outer structure of the substrate. The process is hardly different from that of the untreated substrate, but the amount of biogas generated is increased by +26.341%, reaching a methane ratio of 59.751%.

Lyophilization pretreatment causes a large increase in solubility of the substrate (+98.805%) and causes a huge increase in porosity. However, moisture is drastically reduced (−98.805%), and this leads to a large slowdown in hydrolysis. The increase in the solubility and accessibility of the substrate, coupled with the slow hydrolysis generates a synergy, compensating the release of VFA, eliminating it at a correct rate that ensures that they do not accumulate. Although there are no significant changes in biogas generation, it is more enriched in methane with a ratio of 48.761%.

Among the three studied pretreatments, the one that produces the best results is freezing. Although all three produce increased amounts of biogas, and the methane content is greatly increased in all three cases, in the freezing scenario the disintegration constant is much higher than the constant of the other two processes, with an increase higher than 300%. This makes the pretreatment by freezing the most suitable as it also accelerates the process and increases the level of degradation. In addition, it is the simplest to incorporate in treatment plants.

This study assesses the variations in biogas production in order to evaluate the energetic potential of the process. However there are further limitations that need to be studied or considered, such as the economic viability of the process, taking into account the different existing technologies. Further studies are needed to analyze the economics of the process, its large-scale implementation, and any technical limitations.

## Figures and Tables

**Table 1:** Standardized procedures used for compositional characterization of substrates and mixtures.

Method Procedure	
Proximate Analysis (Physical Parameters)	
Hum [%_hb_]	APHA 2540-G
TS [%_hb_]	
VS [%_hb_]	
Ashes [%_hb_]	
Macronutritional Analysis (LPCH Content)	
Lipids (L) [%_hb_]	UNE-EN-ISO 13804:2013
Proteins (P) [%_hb_]	
Carbohydrates (CH) [%_hb_]	
Organic Content Analysis	
COD_t_ [mg O_2_/g—mL]	APHA 5220-B
COD_f_ [mg O_2_/g—mL]	
Solubility [%]	
Nitrogen Content Analysis	
TKN [mg N/g—mL]	APHA 4500-N
AN [mg N/g—mL]	APHA 4500-NH_3_
ON [mg N/g—mL]	APHA 4500-N_org_
pH and Alkalinity Analysis	
pH	
TA [mg CaCO_3_/g—mL]	APHA 2320-B
PA [mg CaCO_3_/g—mL]	
IA [mg CaCO_3_/g—mL]	
Ultimate Analysis (Elemental Analysis)	
C [%_db_]	UNE-EN-ISO 15104:2011
H [%_db_]	
N [%_db_]	
S [%_db_]	
C/N Ratio	

**Table 2:** Gross production of biogas, methane and hydrogen, and descriptive statistics for the BMP test when digesting V.

	Production [NmL/100 g of Residue V]	Standard Deviation σ	Coefficient of Variation CV	Relative Error ε
Biogas	913.282 NmL	222.904	0.244	14.436%
Methane	289.333 NmL	94.723	0.327	21.421%
Hydrogen	0.456 NmL + 0.200 NmL	0.298 + 0.200	0.655 + 1.000	57.583% + 90.609%

**Table 3:** Methane and hydrogen content of the produced biogas, and descriptive statistics for the BMP test when digesting V.

	Content [% vol]	Standard Deviation σ	Coefficient of Variation CV	Relative Error ε
Methane	32.252%	7.906	0.245	12.051%
Hydrogen	0.265% + 0.017%	0.128 + 0.019	0.484 + 1.124	42.311% + 92.375%

**Table 4:** Results obtained in mathematical processing of the parameters of substrate V biodegradation.

		Standard Deviation σ	Relative Error ε
Theoretical methane generation	292.808 NmL	91.809	22.260%
Maximum methane generation	323.000 NmL	90.961	16.786%
Disintegration constant	0.200 days^−^^1^	0.044	17.920%
Substrate biodegradation	16.045%	1.677	7.422%

**Table 5:** Characterization results for BMP tests of substrate V and pretreated substrate VF at the start and after completion of the BMP test.

	Substrate	Substrate		Initial Reactor Mix		Final Reactor Mix
	V	VF		VF + F		VF + F
Proximate Analysis (Physical parameters)						
Hum [%_hb_]	87.90	87.60	➞	92.61	⇝	89.54
TS [%_hb_]	12.10	12.40		7.40		5.12
VS [%_hb_]	10.91	11.26		6.51		3.05
Macronutritional Analysis (LPCH Content)						
Lipids (L) [%_hb_]	0.48	0.68	➞	0.53	⇝	
Proteins (P) [%_hb_]	1.52	1.92		0.88		
Carbohydrates (CH) [%_hb_]	8.90	8.70		2.61		
Organic Content Analysis (COD)						
COD_t_ [mg O_2_/g—mL]	173.64	175.26	➞	121.02	⇝	113.87
COD_f_ [mg O_2_/g—mL]	41.83	56.11		47.12		11.02
Solubility [%]	24.09	32.02		34.80		9.67
Nitrogen Content Analysis						
TKN [mg N/g—mL]	2.46	2.56	➞	2.14	⇝	2.32
AN [mg N/g—mL]	0.03	0.02		0.87		1.14
ON [mg N/g—mL]	2.43	2.53		1.26		1.12
pH and Alkalinity Analysis						
pH	4.96	4.73	➞	6.77	⇝	7.01
TA [mg CaCO_3_/g—mL]	5.83	2.71		7.45		119.72
PA [mg CaCO_3_/g—mL]	-	-		4.02		3.98
IA [mg CaCO_3_/g—mL]	5.83	2.71		1.23		8.56
Ultimate Analysis (Elemental Analysis)						
C [%_db_]	34.52	36.25	➞	17.42	⇝	
H [%_db_]	6.43	6.75		8.41		
N [%_db_]	1.69	1.77		2.11		
S [%_db_]	0.09	0.10		0.16		
C/N Ratio	20.43	20.43		8.25		

**Table 6:** Gross production of biogas, methane and hydrogen, and descriptive statistics for the BMP test when digesting VF.

	Production [NmL/100 g of Substrate VF]	Standard Deviation σ	Coefficient of Variation CV	Relative Error ε
Biogas	1097.469 NmL	90.913	0.082	6.094%
Methane	651.319 NmL	68.978	0.105	7.790%
Hydrogen	4.066 NmL	1.204	0.296	23.177%

**Table 7:** Methane and hydrogen content of the produced biogas, and descriptive statistics for the BMP test when digesting VF.

	Content [% vol]	Standard Deviation σ	Coefficient of Variation CV	Relative Error ε
Methane	59.438%	5.165	0.086	5.838%
Hydrogen	0.903%	0.233	0.258	20.291%

**Table 8:** Results obtained in mathematical processing of the parameters of substrate VF biodegradation.

		Standard Deviation σ	Relative Error ε
Theoretical methane generation	639.253 NmL	58.469	12.966%
Maximum methane generation	651.319 NmL	57.590	19.422%
Disintegration constant	0.813 days^−1^	0.209	18.423%
Substrate biodegradation	21.565%	2.850	9.598%

**Table 9:** Characterization results for BMP tests of substrate V and pretreated substrate VU at the start and after completion of the BMP test.

	Substrate	Substrate		Initial Reactor Mix		Final Reactor Mix
	V	VU		VU + F		VU + F
Proximate Analysis (Physical parameters)						
Hum [%_hb_]	87.90	87.50	➞	92.60	⇝	84.51
TS [%_hb_]	12.10	12.50		7.40		5.92
VS [%_hb_]	10.91	11.09		6.46		3.28
Macronutritional Analysis (LPCH Content)						
Lipids (L) [%_hb_]	0.48	0.51	➞	0.48	⇝	
Proteins (P) [%_hb_]	1.52	1.62		0.80		
Carbohydrates (CH) [%_hb_]	8.90	8.90		2.64		
Organic Content Analysis (COD)						
COD_t_ [mg O_2_/g—mL]	173.64	171.24	➞	119.04	⇝	113.61
COD_f_ [mg O_2_/g—mL]	41.83	41.00		38.06		13.52
Solubility [%]	24.09	23.94		31.97		11.90
Nitrogen Content Analysis						
TKN [mg N/g—mL]	2.46	2.47	➞	2.12	⇝	2.28
AN [mg N/g—mL]	0.03	0.03		0.87		1.09
ON [mg N/g—mL]	2.43	2.44		1.25		1.19
pH and Alkalinity Analysis						
pH	4.96	4.95	➞	6.83	⇝	6.97
TA [mg CaCO_3_/g—mL]	5.83	4.02		7.65		8.40
PA [mg CaCO_3_/g—mL]	-	-		3.91		6.95
IA [mg CaCO_3_/g—mL]	5.83	4.02		3.74		1.95
Ultimate Analysis (Elemental Analysis)						
C [%_db_]	34.52	33.83	➞	16.85	⇝	
H [%_db_]	6.43	6.30		3.25		
N [%_db_]	1.69	1.66		2.08		
S [%_db_]	0.09	0.09		0.15		
C/N Ratio	20.43	20.43		8.10		

**Table 10:** Gross production of biogas, methane and hydrogen, and descriptive statistics for the BMP test when digesting VU.

	Production [NmL/100 g of Substrate VU]	Standard Deviation σ	Coefficient of Variation CV	Relative Error ε
Biogas	1153.493 NmL	135.690	0.117	8.667%
Methane	690.123 NmL	114.251	0.165	12.220%
Hydrogen	0.627 NmL	0.315	0.503	43.695%

**Table 11:** Methane and hydrogen content of the produced biogas, and descriptive statistics for the BMP test when digesting VU.

	Content [% vol]	Standard Deviation σ	Coefficient of Variation CV	Relative Error ε
Methane	59.751%	6.194	0.103	8.369%
Hydrogen	0.755%	0.327	0.437	39.911%

**Table 12:** Results obtained in mathematical processing of the parameters of substrate VU biodegradation.

		Standard Deviation σ	Relative Error ε
Theoretical methane generation	674.222 NmL	88.176	16.826%
Maximum methane generation	690.123 NmL	87.141	16.393%
Disintegration constant	0.285 days^−1^	0.209	53.236%
Substrate biodegradation	19.856%	1.46	7.422%

**Table 13:** Characterization results for BMP tests of substrate V and pretreated substrate VU at the start and after completion of the BMP test.

	Substrate	Substrate		Initial Reactor Mix		Final Reactor Mix
	V	VL		VL + F		VL + F
Proximate Analysis (Physical parameters)						
Hum [%_hb_]	87.90	1.05	➞	70.14	⇝	69.52
TS [%_hb_]	12.10	9.92		6.77		5.81
VS [%_hb_]	10.91	8.97		5.94		3.48
Macronutritional Analysis (LPCH Content)						
Lipids (L) [%_hb_]	0.48	0.44	➞	0.39	⇝	
Proteins (P) [%_hb_]	1.52	1.07		0.77		
Carbohydrates (CH) [%_hb_]	8.90	7.81		2.35		
Organic Content Analysis (COD)						
COD_t_ [mg O_2_/g—mL]	173.64	135.26	➞	110.05	⇝	105.99
COD_f_ [mg O_2_/g—mL]	41.83	64.12		39.02		14.51
Solubility [%]	24.09	47.40		35.46		13.69
**Nitrogen Content Analysis**						
TKN [mg N/g—mL]	2.46	2.02	➞	2.02	⇝	2.27
AN [mg N/g—mL]	0.03	0.02		1.16		1.44
ON [mg N/g—mL]	2.43	2.00		0.85		0.83
**pH and Alkalinity Analysis**						
pH	4.96	4.95	➞	6.81	⇝	6.84
TA [mg CaCO_3_/g—mL]	5.83	4.15		7.54		8.40
PA [mg CaCO_3_/g—mL]	-	-		3.85		6.85
IA [mg CaCO_3_/g—mL]	5.83	4.15		3.69		2.05
**Ultimate Analysis (Elemental Analysis)**						
C [%_db_]	34.52	33.87	➞	16.74	⇝	
H [%_db_]	6.43	6.21		8.30		
N [%_db_]	1.69	1.63		2.07		
S [%_db_]	0.09	0.08		0.15		
C/N Ratio	20.43	20.43		8.09		

**Table 14:** Gross production of biogas, methane and hydrogen, and descriptive statistics for the BMP test when digesting VL.

	Production [NmL/100 g of Substrate VL]	Standard Deviation σ	Coefficient of Variation CV	Relative Error ε
Biogas	1046.492 NmL	131.253	0.125	8.564%
Methane	511.657 NmL	90.442	0.176	13.333%
Hydrogen	1.085 NmL	0.376	0.346	24.188%

**Table 15:** Methane and hydrogen content of the produced biogas, and descriptive statistics for the BMP test when digesting VL.

	Content [% vol]	Standard Deviation σ	Coefficient of Variation CV	Relative Error ε
Methane	48.761%	5.433	0.111	8.359%
Hydrogen	0.183%	0.050	0.274	20.487%

**Table 16:** Results obtained in mathematical processing of the parameters of substrate VL biodegradation.

		Standard Deviation σ	Relative Error ε
Theoretical methane generation	523.287 NmL	44.287	18.821%
Maximum methane generation	576.720 NmL	42.515	19.108%
Disintegration constant	0.129 days^−1^	0.231	50.389%
Substrate biodegradation	20.907%	3.444	9.668%

**Table 17:** Summary of the effect pretreatments compared to the substrate without pretreatment.

		V	VF		VU		VL	
				Increase (%)		Increase (%)		Increase (%)
**Biogas**	**Production** [NmL/100 g of substrate]	913.282 NmL	1097.469 NmL	+20.200%	1153.493 NmL	+26.341%	1046.492 NmL	+14.621%
**Methane**	**Production** [NmL/100 g of substrate]	289.333 NmL	651.319 NmL	+125.370%	690.123 NmL	+138.797%	511.657 NmL	+77.044%
	**Content** [%vol biogas]	32.252%	59.438%	+85.743%	59.751%	+86.612%	48.761%	+52.378%
**Hydrogen**	**Production** [NmL/100 g of substrate]	0.456 + 0.200 NmL	4.066 NmL	+790%	0.627 NmL	+37.500%	1.085 NmL	+137.938%
	**Content** [%vol biogas]	0.265 + 0.017%	0.903%	240.754%	0.755%	+184.905%	0.183%	−30.943%

## List of Acronyms

AD	Anaerobic Digestion
AN	Ammoniacal Nitrogen
ANOVA	Analysis of Variance
BD	Biodegradation
BMP	Biochemical Methane Potential
C/N	Carbon/Nitrogen Ratio
COD	Chemical Oxygen Demand
CODf	Chemical Oxigen Demand (filtered or soluble fraction)
CODt	Chemical Oxigen Demand (total fraction)
CV	Coefficient of variation
FBP	Food By-Product
FL	Food Loose
FSC	Food Supply Chain
FW	Food Waste
GC	Gas Chromatograph
Hum	Humidity
IA	Intermediate Alkalinity
k_dis_	Disintegration constant
LCFA	Long Chain Fatty Acid
LPCH	Lipids, Proteins and Carbohydrates content
OM	Organic Matter
ON	Organic Nitrogen
PA	Partial Alkalinity
S	Sludge (Inoculum)
TA	Total Alkalinity
TKN	Total Kjeldahl Nitrogen
TS	Total Solids
UASB	Upflow Anaerobic Sludge Blanket reactor
VFA	Volatile Fatty Acid
VS	Volatile Solids
WWTP	Wastewater Treatment Plant

## References

[1] Eurostat Fooddrink Europe (2019). Economic Bulletin Q1 2019.

[2] Lipinski B., Hanson C., Lomax J., Kitinoja L., Waite R., Searchinger T. (2013). Reducing food loss and waste. World Resources Institude Working Paper.

[3] Buzby J.C., Hyman J. (2012). Total and per capita value of food loss in the United States. Food Policy.

[4] Kojima R., Ishikawa M. (2013). Prevention and Recycling of Food Wastes in Japan: Policies and Achievements. http://www.lib.kobe-u.ac.jp/repository/81005266.pdf.

[5] European Commision Health and Food Safety. https://ec.europa.eu/info/departments/health-and-food-safety_en.

[6] Monier V., Mudgal S., Escalon V., O’Connor C., Gibon T., Anderson G., Montoux H., Reisinger H., Dolley P., Ogilvie S. (2010). Preparatory Study on Food Waste across EU 27.

[7] Bräutigam K.-R., Jörissen J., Priefer C. (2014). The extent of food waste generation across EU-27: Different calculation methods and the reliability of their results. Waste Manag. Res..

[8] Nellman C., MacDevette M., Manders T., Eickhout B., Svihus B., Prins A. (2009). The Environmental Food Crisis—The Environment’s Role in Averting Future Food Crises.

[9] Tuck C.O., Perez E., Horvath I.T., Sheldon R.A., Poliakoff M. (2012). Valorization of biomass: Deriving more value from waste. Science.

[10] Sanders J., Scott E., Weusthuis R., Mooibroek H. (2007). Bio-refinery as the bio-inspired process to bulk chemicals. Macromol. Biosci..

[11] Sakai K., Ezaki Y. (2006). Open L-lactic acid fermentation of food refuse using thermophilic Bacillus coagulans and fluorescence in situ hybridization analysis of microflora. J. Biosci. Bioeng..

[12] Wang Q., Wang X., Wang X., Ma H., Ren N. (2005). Bioconversion of kitchen garbage to lactic acid by two wild strains of *Lactobacillus* species. J. Environ. Sci. Health Part A.

[13] Yang S.Y., Ji K.S., Baik Y.H., Kwak W.S., McCaskey T.A. (2006). Lactic acid fermentation of food waste for swine feed. Bioresour. Technol..

[14] Koike Y., An M.-Z., Tang Y.-Q., Syo T., Osaka N., Morimura S., Kida K. (2009). Production of fuel ethanol and methane from garbage by high-efficiency two-stage fermentation process. J. Biosci. Bioeng..

[15] Rao M. (2004). Bioenergy conversion studies of organic fraction of MSW: Kinetic studies and gas yield?organic loading relationships for process optimisation. Bioresour. Technol..

[16] Zhang C., Xiao G., Peng L., Su H., Tan T. (2013). The anaerobic co-digestion of food waste and cattle manure. Bioresour. Technol..

[17] Kim J.K., Oh B.R., Shin H.-J., Eom C.-Y., Kim S.W. (2008). Statistical optimization of enzymatic saccharification and ethanol fermentation using food waste. Process Biochem..

[18] Tang Y.-Q., Koike Y., Liu K., An M.-Z., Morimura S., Wu X.-L., Kida K. (2008). Ethanol production from kitchen waste using the flocculating yeast Saccharomyces cerevisiae strain KF-7. Biomass Bioenergy.

[19] Alptekin E., Canakci M., Sanli H. (2014). Biodiesel production from vegetable oil and waste animal fats in a pilot plant. Waste Manag..

[20] Han S. (2004). Biohydrogen production by anaerobic fermentation of food waste. Int. J. Hydrog. Energy.

[21] Pan J., Zhang R., Elmashad H., Sun H., Ying Y. (2008). Effect of food to microorganism ratio on biohydrogen production from food waste via anaerobic fermentation. Int. J. Hydrog. Energy.

[22] Morales-Polo C., Cledera-Castro M.D.M., Soria B.Y.M. (2019). Biogas production from vegetable and fruit markets waste—Compositional and batch characterizations. Sustainability.

[23] Gujer W., Zehnder A.J.R. (1983). Conversion processes in anaerobic digestion. Water Sci. Technol..

[24] Hawkes F.R. (1980). The biochemistry of anaerobic digestion. Biomethane: Production and Uses.

[25] Veeken A., Hamelers B. (1999). Effect of temperature on hydrolysis rates of selected biowaste components. Bioresour. Technol..

[26] Zhang C., Su H., Baeyens J., Tan T. (2014). Reviewing the anaerobic digestion of food waste for biogas production. Renew. Sustain. Energy Rev..

[27] Kwietniewska E., Tys J. (2014). Process characteristics, inhibition factors and methane yields of anaerobic digestion process, with particular focus on microalgal biomass fermentation. Renew. Sustain. Energy Rev..

[28] Khalid A., Arshad M., Anjum M., Mahmood T., Dawson L. (2011). The anaerobic digestion of solid organic waste. Waste Manag..

[29] Agyeman F.O., Tao W. (2014). Anaerobic co-digestion of food waste and dairy manure: Effects of food waste particle size and organic loading rate. J. Environ. Manag..

[30] Palmowski L.M., Müller J.A., Palmowski L.M., Müller J.A. (2000). Influence of the size reduction of organic waste on their anaerobic digestion. Water Sci. Technol..

[31] Cesaro A., Naddeo V., Amodio V., Belgiorno V. (2012). Enhanced biogas production from anaerobic codigestion of solid waste by sonolysis. Ultrason. Sonochem..

[32] Zhang Z., Zhang L., Zhou Y., Chen J., Liang Y., Wei L. (2013). Pilot-scale operation of enhanced anaerobic digestion of nutrient-deficient municipal sludge by ultrasonic pretreatment and co-digestion of kitchen garbage. J. Environ. Chem. Eng..

[33] Izumi K., Okishio Y., Nagao N., Niwa C., Yamamoto S., Toda T. (2010). Effects of particle size on anaerobic digestion of food waste. Int. Biodeterior. Biodegrad..

[34] Schattauer A., Abdoun E., Weiland P., Plöchl M., Heiermann M. (2011). Abundance of trace elements in demonstration biogas plants. Biosyst. Eng..

[35] Yang D., Wei S.J., Wen Q.M., Zhang X.J. (2014). Comparison of pretreatments for lignocellulosic biomass. Adv. Mater. Res..

[36] Li Y., Jin Y. (2015). Effects of thermal pretreatment on acidification phase during two-phase batch anaerobic digestion of kitchen waste. Renew. Energy.

[37] Elbeshbishy E., Nakhla G. (2011). Comparative study of the effect of ultrasonication on the anaerobic biodegradability of food waste in single and two-stage systems. Bioresour. Technol..

[38] Chiu S.L.H., Lo I.M.C. (2016). Reviewing the anaerobic digestion and co-digestion process of food waste from the perspectives on biogas production performance and environmental impacts. Environ. Sci. Pollut. Res..

[39] Ma J., Duong T.H., Smits M., Verstraete W., Carballa M. (2011). Enhanced biomethanation of kitchen waste by different pre-treatments. Bioresour. Technol..

[40] Parfitt J., Barthel M., Macnaughton S. (2010). Food waste within food supply chains: Quantification and potential for change to 2050. Philos. Trans. R. Soc. B Biol. Sci..

[41] Li X., Chen Y., Zhao S., Chen H., Zheng X., Luo J., Liu Y. (2015). Efficient production of optically pure l -lactic acid from food waste at ambient temperature by regulating key enzyme activity. Water Res..

[42] Hassan M.A., Yee L.-N., Yee P.L., Ariffin H., Raha A.R., Shirai Y., Sudesh K. (2013). Sustainable production of polyhydroxyalkanoates from renewable oil-palm biomass. Biomass Bioenergy.

[43] Gasparatos A., Stromberg P., Takeuchi K. (2011). Biofuels, ecosystem services and human wellbeing: Putting biofuels in the ecosystem services narrative. Agric. Ecosyst. Environ..

[44] AENOR (1999). UNE-EN ISO 11734:1999 Water Quality—Evaluation of the “Ultimate” Anaerobic Biodegradability of Organic Compounds in Digested Sludge—Method by Measurement of the Biogas Production. https://www.iso.org/standard/19656.html.

[45] VDI (2016). VDI 4630 Fermentation of Organic Materials. Characterisation of the Substrate, Sampling, Collection of Material Data, Fermentation Tests. https://www.vdi.de/richtlinien/details/vdi-4630-fermentation-of-organic-materials-characterization-of-the-substrate-sampling-collection-of-material-data-fermentation-tests.

[46] APHA, AWWA, WEF (2005). Standard Methods for the Examination of Water and Wastewater.

[47] Gutiérrez M.E. (2014). Co-Digestión Anaerobia de Lodo de Edar Con Residuos Orgánicos de Diferente Naturaleza: Combinación de Técnicas Experimentales y Herramientas Matemáticas.

